# *Gb_ANR-47* Enhances the Resistance of *Gossypium barbadense* to *Fusarium oxysporum* f. sp. *vasinfectum* (FOV) by Regulating the Content of Proanthocyanidins

**DOI:** 10.3390/plants11151902

**Published:** 2022-07-22

**Authors:** Xuening Su, Jieyin Zhao, Wenju Gao, Qianli Zu, Quanjia Chen, Chunping Li, Yanying Qu

**Affiliations:** 1 Engineering Research Centre of Cotton, Ministry of Education/College of Agriculture, Xinjiang Agricultural University, 311 Nongda East Road, Urumqi 830052, China; sue1114@126.com (X.S.); cottonzjy@126.com (J.Z.); gaowenju588@126.com (W.G.); xjzuqianli@126.com (Q.Z.); chqjia@126.com (Q.C.); 2 Institute of Cash Crops, Xinjiang Academy of Agricultural Sciences, Urumqi 830052, China

**Keywords:** *G. barbadense*, proanthocyanidins, FOV, *Gb_ANR-47*

## Abstract

Anthocyanidin reductase (ANR) is an important regulator of flavonoid metabolism, and proanthocyanidins, the secondary metabolites of flavonoids, play an important role in the response of plants to pathogenic stress. Therefore, in this study, the expression analysis of the ANR gene family of *Gossypium barbadense* after inoculation with *Fusarium oxysporum* f. sp. *vasinfectum* (FOV) was performed at different time points. It was found that *Gb_ANR-47* showed significant differences in the disease-resistant cultivar 06-146 and the susceptible cultivar Xinhai 14, as well as in the highest root expression. It was found that the expression of *Gb_ANR-47* in the resistant cultivar was significantly higher than that in the susceptible cultivar by MeJA and SA, and different amounts of methyl jasmonate (MeJA) and salicylic acid (SA) response elements were found in the promoter region of *Gb_ANR-47*. After silencing *GbANR-47* in 06-146 material by VIGS technology, its resistance to FOV decreased significantly. The disease severity index (DSI) was significantly increased, and the anthocyanin content was significantly decreased in silenced plants, compared to controls. Our findings suggest that *GbANR-47* is a positive regulator of FOV resistance in *Gossypium barbadense*. The research results provide an important theoretical basis for in-depth analysis of the molecular mechanism of *GbANR-47* and improving the anti-FOV of *Gossypium barbadense*.

## 1. Introduction

In China, island cotton (*G. barbadense*) is mainly planted in the southern area of Xinjiang [1]. *Fusarium oxysporum* f. sp. *vasinfectum* (FOV) is currently the most severe disease affecting island cotton production [2]. This disease is caused by the soil-borne plant fungal pathogen *F.* *oxysporum*, which exists as mycelia or small sclerotia in soil and crop residue [3]. Normally, this pathogen can survive for 5 to 10 years and can start infecting plants at the seedling stage [3,4]. Especially after infection at the seedling stage, leaf chlorosis, necrosis, withering, and discoloration of vascular tissue appear, resulting in a reduction in cotton bolls and boll weight [4]. In severe cases, plant death and decreases in cotton production and fiber quality can occur [3,4,5].

Flavonoids are secondary metabolites present in plants [6]. They are plant polyphenols with the general structure of a 15-carbon skeleton arranged in two phenyl rings and a heterocyclic ring which can exist as a free aglycone or form glycosides [7,8,9]. In plants, the complex structural diversity of flavonoids determines the diversity of their functions, such as protection against UV irradiation, insect pests, diseases, and other biotic and abiotic stresses [10,11,12]. Proanthocyanidins are synthesized from anthocyanidins via catalysis of anthocyanidin reductase (ANR) and are secondary metabolites that function downstream of the flavonoid metabolic pathway, in which ANR genes play an important role [13]. Overexpression of ANR genes from rose has been shown to increase tolerance to abiotic stress by increasing reactive oxygen species (ROS) clearance and by regulating abscisic acid (ABA) signaling in tobacco [14]. Plate antibacterial tests of transgenic tobacco extracts have further confirmed the inhibitory effect of flavonoids on bacterial and fungal growth [15].

Previous studies have shown that both allotetraploid island cotton and upland cotton evolved directly through hybridization and doubling of the A and D genomes. *Gossypium* *arboreum* (A2 genome) is the donor of the A genome, and *G. raimondii* (D5 genome) is the donor of the D genome [16,17,18,19]. The biosynthesis of flavonoids is mainly achieved through the phenylpropane metabolic pathway [20]. The starting substrates 4-coumaroyl-CoA and malonyl-CoA are formed by chalcone synthase (CHS) to form chalcone, which is then catalyzed by chalcone isomerase to generate 4, 5, 7-trihydroxyl flavanones, which then enter other metabolic pathways to form different flavonoids. ANR can catalyze the oxidation of colorless procyanidins to produce colored anthocyanins [20]. The ANR pathway is involved in proanthocyanidin biosynthesis, as demonstrated by enzymatic analysis, ectopic expression, and gene silencing experiments in *G. hirsutum* [20]. However, studies of the ANR gene in *G. barbadense* have not been reported. Therefore, a comprehensive and systematic analysis of the ANR gene family was conducted to explore the phylogeny and evolution of ANR genes and to determine the expression profile of the ANR gene family members in cotton under FOV stress. In this study, 69 ANR genes were identified. Phylogenetic analysis was performed to elucidate the evolution and function of the ANR genes in *G.* *barbadense*, and their physical location and gene structure were determined. Moreover, expression analysis and virus-induced gene silencing (VIGS) revealed a potential anti-FOV function of *Gb_ANR-47* in *G.* *barbadense*. These results will improve our understanding of ANR genes in plants and lay a foundation for subsequent analysis of ANR gene molecular mechanisms.

## 2. Results

### 2.1. Identification of the ANR Gene Family Members in Cotton

To systematically study the copy number changes that occurred in the ANR gene family during cotton evolution, all of the ANR gene sequences were retrieved from *G. arboreum*, *G. raimondii*, *G. hirsutum*, and *G. barbadense* by using the protein sequences of Arabidopsis and rice. The search results were validated via the NCBI-CDD database (Figure S1). In total, 35, 37, 68, and 69 sequences were detected in *G. arboreum*, *G. raimondii*, *G. hirsutum*, and *G. barbadense*, respectively. GbANR-1~GbANR-69 were named according to their position on the chromosome in cotton. The length of the open reading frame (ORF) of the ANR family genes ranges from 306 to 1899 bp, and 101 to 632 amino acid residues compose the encoded proteins; the relative molecular mass is between 10.96 and 70.34 kDa. The theoretical isoelectric points range from 4.92 to 9.22. Protein subcellular localization predicted that 45 proteins were localized to the cytoplasm, three were localized to the chloroplast, eight to the nucleus, seven to the intrinsic system, two to the extracellular system, two to organellar membranes, one to the mitochondrion, and one to the plasma membrane (Table 1).

**Figure 1:**
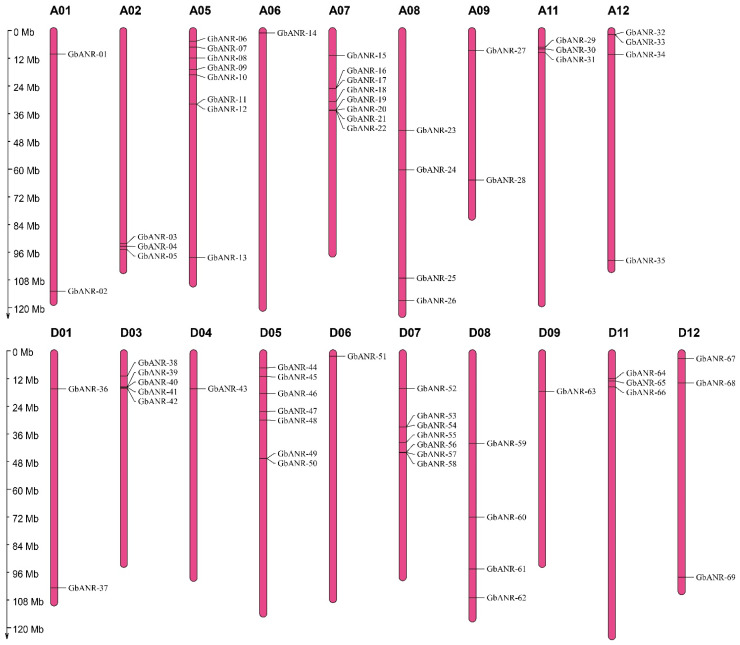
Chromosomal localization of *G. barbadense* ANR genes.

Sixty-nine GbANR genes were distributed on 19 chromosomes (A01, A02, A05, A06, A07, A08, A09, A11, A12, D01, D03, D04, D05, D06, D07, D08, D09, D11, and D12) (Figure 1). Thirty-five ANR genes and thirty-four ANR genes were present in subgroup A and subgroup D, respectively. Previous studies have suggested that *G. arboreum* and *G. raimondii* are donor species of the A subgenome and D subgenome, respectively, of *G. barbadense*. The number of ANR genes in subgroup A in *G. barbadense* was consistent with that in *G. arboreum*. Moreover, the number of ANR genes in subgroup D was three less than the number of ANR genes in *G. raimondii*. This indicates that the loss of ANR genes may have occurred due to redundancy in gene function. There are three ANR genes on chromosome A02. However, there were none on D02, A03, or A04, while D03 and D04 contained five and one ANR genes, respectively, in *G. barbadense*, which shows that ANR genes may have been lost but duplicated during the course of evolution. Overall, subgroup A still has a strong correspondence with subgroup D, which is also consistent with the evolutionary relationship results for cotton.

### 2.2. Evolutionary Analysis of the ANR Genes in Cotton

**Figure 2:**
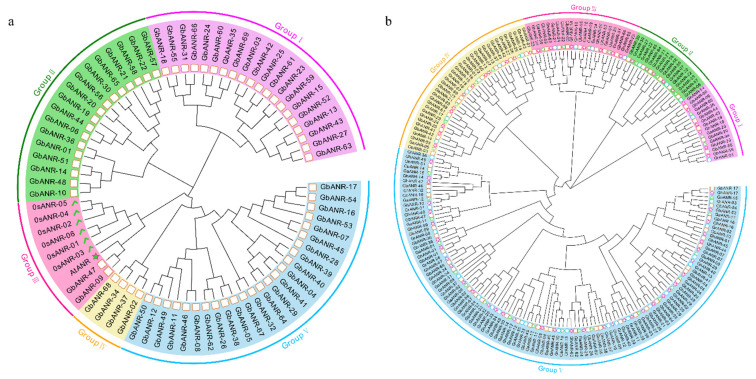
(**a**) Phylogenetic tree of *Arabidopsis thaliana*, rice, and *G. barbadense*. Squares represent ANR genes of *G. barbadense*, five-pointed stars represent ANR genes of *Arabidopsis thaliana*, and ticks represent ANR genes of rice. (**b**) Phylogenetic tree of *G. arboreum, G. raimondii, G. hirsutum*, and *G. barbadense*. The five-pointed star represents the ANR gene of *G. hirsutum*, the square represents the ANR gene of *G. barbadense*, the triangle represents the ANR gene of *G. arboreum*, and the circle represents the ANR gene of *G. raimondii*.

To further understand the evolutionary relationship of the ANR genes in cotton, a phylogenetic tree including the full length of 69 ANR genes from *G. barbadense*, six ANR genes from rice, and one ANR gene sequence from *Arabidopsis thaliana* was constructed (Figure 2a). The tree was divided into five groups according to the results. Only subgroup III contained ANR genes from *Arabidopsis thaliana* and rice, suggesting that significant tandem duplication of the ANR gene family members occurred during the course of evolution, resulting in an increased amount of ANR genes in tetraploid cotton.

To explore the phylogenetic relationships of the ANR gene family members in cotton, an evolutionary tree was constructed using ANR gene protein sequences from four different cotton species (Figure 2b). The number of ANR genes in each subgroup was basically twice that in *G. arboreum* and *G. raimondii*. This result is consistent with the results of the previous analysis, which is in line with the evolutionary relationship findings in cotton. This indicates that the ANR family genes have been relatively conserved during their evolution in cotton. Although there were relatively few members in groups I, II, and III, they have persisted throughout the evolution of cotton, suggesting that they may play important roles in biological processes.

### 2.3. Evolutionary Tree, Gene Structure, and Motif Analysis of Cotton ANR Genes

**Figure 3:**
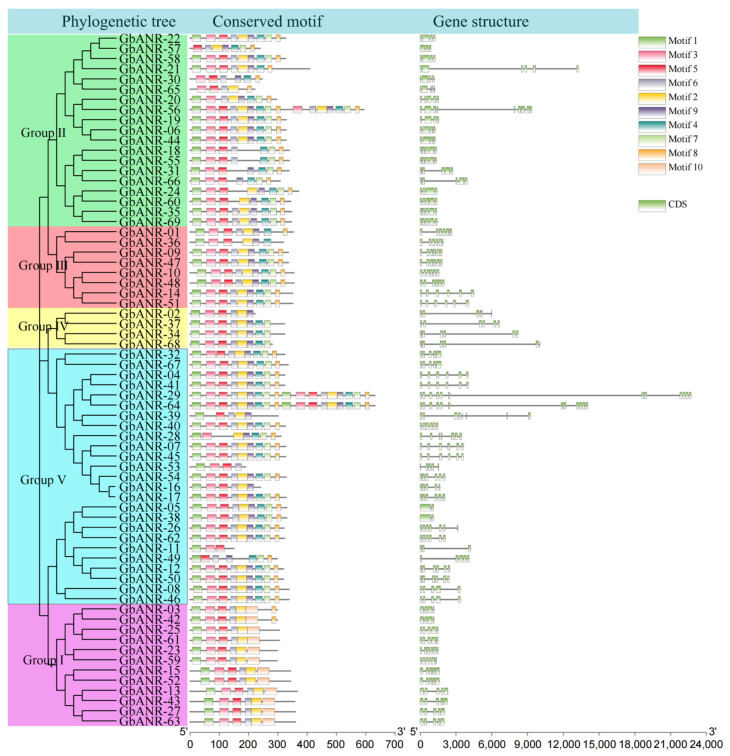
Evolutionary tree, gene structure, and conserved motif analysis of the *G. barbadense* ANR gene family.

An evolutionary tree and both gene structure and motif analyses were constructed and performed, respectively, on the basis of the full-length ANR gene sequences, coding DNA sequences (CDSs), and protein sequences in cotton (Figure 3 and Figure S2). All members of the ANR family could be divided into five subgroups in cotton based on the results of the evolutionary tree. Most of the genes contained the same motifs (motifs 2, 3, 5–7) and five CDS regions, of which only subgroup I contained motif 10. In addition, all members except those of subgroup I contain the same motif (motifs 1 – 9). These results indicated that the ANR gene family can be divided into two categories, with subgroup I being one group and the others composing another group. *GbANR-29* and *GbANR-64* had the most motifs (motifs 1 – 9) and were associated with 12 CDSs. This indicated that the difference in the *GbANR-29* and *GbANR-64* gene structures may be due to changes in gene function or to genome annotation error. *GbANR-56* contained six duplicate motifs (motifs 2, 4, 6 – 9), while the *GbANR-21* gene, which is longer than *GbANR-56*, lacked duplicate motifs. Although *GbANR-39* is a long gene, it contains very few motifs. These results suggested that gene structure and gene length may not be directly related for some genes.

### 2.4. Analysis of Cis-Acting Elements of ANR Genes

**Figure 4:**
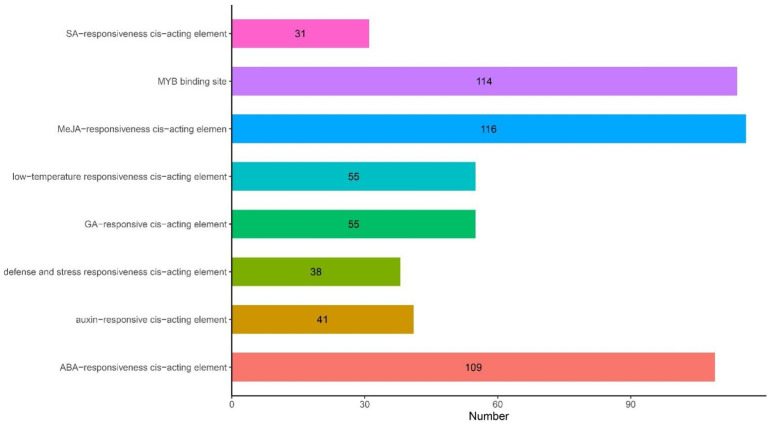
Quantitative analysis of the cis-acting elements in the promoter regions of *G. barbadense* ANR family genes.

Transcription factors (TFs) regulate plant growth and development and response to stress by regulating gene expression, including responses to hormones and environmental factors, cell differentiation, and organ development. Cis-acting element analysis revealed different numbers of cis-acting elements related to phytohormones and responses to environmental stress in the promoter region of the ANR genes in cotton (Figure 4 and Table S1). These mainly included cis-acting elements involved in the defense and stress responses; those involving salicylic acid, ABA, gibberellins, auxin, and jasmonic acid (JA); and those with MYB-binding sites. Each ANR gene promoter contains different numbers and types of cis-acting elements in *G. barbadense*, indicating that they may participate in different biotic and abiotic stress responses through different signaling pathways. Most cis-acting elements were associated with methyl jasmonate (MeJA), revealing that the ANR family may exert its biological functions mainly by functioning synergistically with genes involved in MeJA biosynthesis or signal transduction.

### 2.5. ANR Gene Expression Analysis of G. barbadense

**Figure 5:**
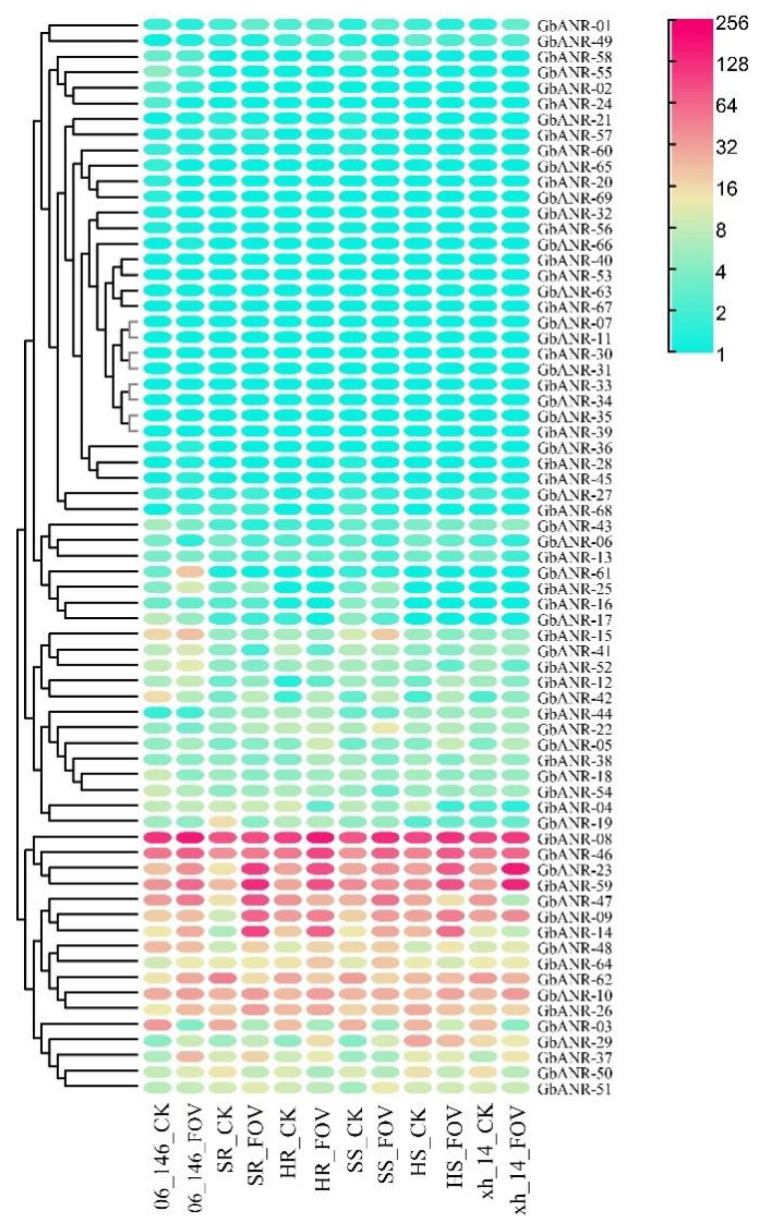
Expression analysis of the ANR genes in *G. barbadense*. The expression level was transformed by log10 (TPM+1). SR (resistant), HR (highly resistant), SS (susceptible), HS (highly susceptible).

In this study, the root transcriptomic data of Xin Hai 14 and 06-146 were analyzed at 40 h post-inoculation (Figure 5). The results showed that the expression of nearly half of the GbANR genes did not change significantly before and after infection; this was the case for *GbANR-03, GbANR-09, GbANR-10, GbANR-14, GbANR-23, GbANR-26, GbANR-29, GbANR-37, GbANR-47, GbANR-48, GbANR-50, GbANR-51, GbANR-59, GbANR-62*, and *GbANR-64*. This suggested that these 15 genes may play an important role in the process of FOV resistance in *G. barbadense*.

### 2.6. qRT–PCR

**Figure 6:**
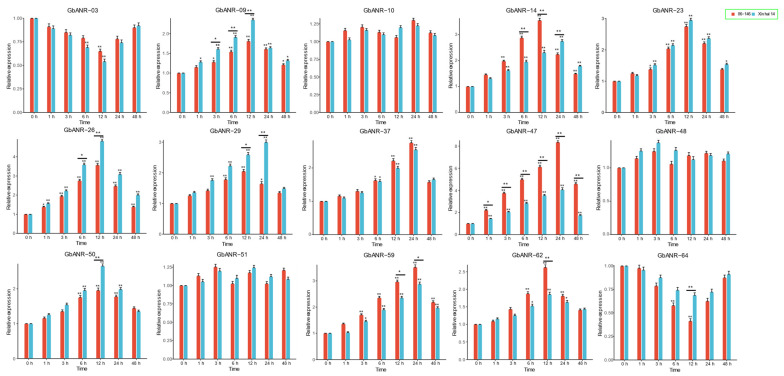
Expression analysis of the ANR gene in *G. barbadense* under FOV. The error bars represent the average of three replicates ± SEs. The difference from the control group is statistically significant at *p* < 0.05 (*) and *p* < 0.01 (**).

Pathogen stress can lead to reprogramming events in the transcriptome, causing changes in metabolite abundance and subsequently affecting plant disease resistance. RNA-seq analysis showed that most of the GbANR genes showed significant expression under FOV stress. Based on the transcriptome expression profiles, we speculated that 15 genes (*GbANR-03*, *GbANR-09*, *GbANR-1*, *GbANR-10*, *GbANR-14*, *GbANR-23*, *GbANR-26*, *GbANR-29*, *GbANR-37*, *GbANR-47*, *GbANR-48*, *GbANR-50*, *GbANR-51*, *GbANR-59*, *GbANR-62*, and *GbANR-64*) may be involved in the resistance to FOV stress in *G. barbadense*. The expression patterns of these 15 genes under FOV stress were further investigated by qRT–PCR in a disease-resistant cultivar (06-146) and a susceptible cultivar (Xin Hai 14). Before inoculation, except for three genes (*GbANR 10, GbANR-40,* and *GbANR-51*), all the genes showed significant changes at different time points after FOV stress (Figure 6). This result suggested that these genes may be involved in the process of FOV residence in cotton. Among these genes, nine (*GbANR-09*, *GbANR-14*, *GbANR-26*, *GbANR-29*, *GbANR-47*, *GbANR-50*, *GbANR-59*, *GbANR-62*, and *GbANR-64*) also showed significant expression at the same time points in the resistant materials. However, only *GbANR-47* was significantly induced at any time point after FOV treatment, and the expression level of this gene in the resistant material was significantly higher than that in the susceptible cultivar. Taken together, these results indicated that *GbANR-47* has an important role in FOV resistance.

**Figure 7:**
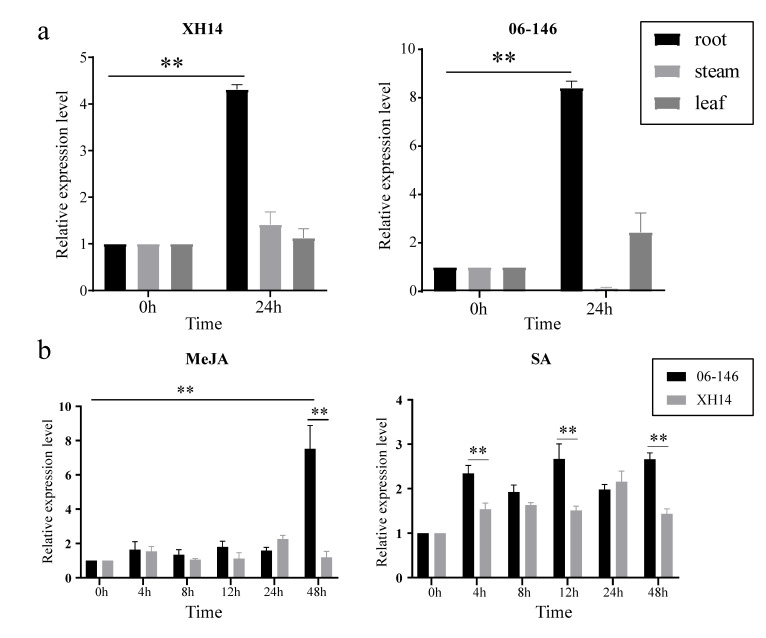
(**a**) Expression analysis of *GbANR-47* in roots, stems and leaves of 06-146 and Xinhai 14 at 24 h and 0 h under FOV stress. (**b**) Expression analysis of GbANR-47 in leaves of 06-146 and Xinhai 14 treated with MeJA and SA at different time points. Error bars represent the average of three replicates ± SE. The difference from the control group is statistically significant, ** *p* < 0.01.

Due to the different parts of gene expression, the molecular biological functions performed are also different. When cotton is invaded by FOV, its initial sensing site is the root. Therefore, we chose to further verify the tissue-specific expression of the *GbANR-47* gene in the two materials before and 24 h after inoculation (Figure 7a). In both resistant and susceptible cultivar, *GbANR-47* was significantly increased in roots, and there was no significant change in stems and leaves. Previous studies have shown that SA and MeJA are important hormones in plant immunity. Therefore, SA and MeJA were selected for inducible expression analysis of *GbANR-47* (Figure 7b). Under the induction of MeJA and SA, the expression of *GbANR-47* in 06-146 was significantly higher than that in Xinhai 14, but SA could induce expression at 4 h after inoculation, while MeJA could significantly induce expression at 48 h. This suggests that *GbANR-47* may play different roles in the MeJA and SA pathways.

### 2.7. VIGS

**Figure 8:**
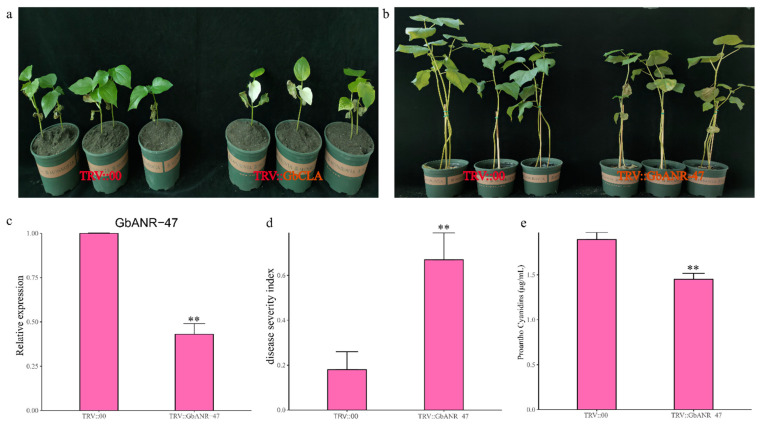
Effects of silencing *Gb_ANR-47* on 06-146 susceptibility to FOV. Two weeks after infiltration, seedlings were inoculated with FOV. (**a**) Ten-day-old cotton plants were infiltrated with Agrobacterium carrying TRV::*GbCLA*. Images were taken at 2 weeks after infiltration; (**b**) representative seedlings of control and silenced plants after inoculation with FOV at 21 days post-inoculation (dpi); (**c**) qRT–PCR results of silencing efficiency; (**d**) responses of control (TRV::00) and silenced (TRV::*Gb_ANR-47*) plants to FOV at 21 dpi. DSI of control and silenced plants, each involving 15 experimental replicates. The DSI was measured at 21 dpi. (**e**) The error bars represent the standard deviations of 15 biological replicates; the asterisks indicate statistically significant differences, as determined by t tests (** *p* < 0.01).

To validate the function of of *GbANR-47* and lay a foundation for elucidating the molecular mechanism underlying its involvement in resistance to FOV, VIGS technology was used. An albino phenotype was observed on newly developed true leaves at 12 days post-injection of plants carrying TRV1 + *GbCLA*, indicating that the VIGS system is reliable (Figure 8a). Also, qRT–PCR analysis showed that *GbANR-47* expression was significantly lower in the virus-induced gene-silenced plants than in control plants harboring an empty vector (Figure 8c). To further understand the relationship between changes in *GbANR47* expression and FOV resistance in virus-induced gene-silenced plants, FOV resistance was evaluated in the virus-induced gene-silenced plants (Figure 8b,d). After inoculation, the virus-induced gene-silenced plants showed more leaf yellowing and necrosis compared to those of the plants in which TRV::00 was silenced (Figure 8c), with a significantly higher DSI for the former (Figure 8d). The ANR gene is an important regulator of proanthocyanidin synthesis, which plays an important role in the process of disease resistance in plants. Therefore, we measured the proanthocyanidin contents of the virus-induced gene silenced plants and TRV::00-silenced plants and found that they decreased significantly in the *GbANR-47*-silenced plants compared with the TRV::00-silenced plants (Figure 8e). Inhibition of *GbANR-47* gene expression in the disease-resistant cultivar, 06-146, resulted in an increased sensitivity to bacterial wilt disease. Together, our results suggested that *GbANR-47* is a positive regulator of FOV resistance in *G. barbadense*.

## 3. Discussion

The ANR gene family plays important roles in plant growth and development and stress resistance [13,21,22,23]. However, few studies have been conducted on the ANR gene family in plants. One ANR protein have been found in *Arabidopsis thaliana*, and six ANR proteins have been found in rice; however, there are fewer reports on ANR proteins in other species. Along with the development of sequencing technology, through genome-wide analysis, it has become possible to identify the origin, diversity, and biological functions of gene families [24]. Studies on ANR family genes in cotton are rare. This study was the first to reveal that there are more ANR family genes in cotton than in rice and *Arabidopsis thaliana*, which may be the result of the doubling of the genome in cotton. According to gene number differences, phylogenetics, and chromosomal locations of the ANR gene family members in cotton, the ANR gene family has been conserved throughout the long-term evolution of cotton. Although A01 has the longest chromosome length, it contains only two ANR genes, which indicates that there is no correlation between the distribution of ANR genes in *G. barbadense* and the chromosome length (Figure 1).

Protein structure and gene structure are closely related to gene function [25]. Most genes in the same subgroup of an evolutionary tree have the same or similar gene structure and motif structure, which is consistent with the structures found for model plant species [26]. In general, gene function may be altered due to alterations in exon/intron structures or conserved domains [27]. It was also found in the ANR gene family in *G. barbadense* that most of the genes contained the same motifs and generally contained five CDS regions, among which only subgroup I members contained motif 10. This indicates that the ANR gene family can be divided into two categories according to whether they contain motif 10. One set contains motif 10, and the other set does not. *GbANR-56* contains six duplicate motifs (motifs 2, 4, 6 – 9), while the *GbANR-21* gene, which is longer than *GbANR-56*, lacks any duplicate motif. *GbANR-39* is a long gene but contains very few motifs. These findings indicated that gene structure and gene length may not be directly related in the case of some genes.

TFs regulate plant growth and development and stress resistance by regulating gene expression, mainly including genes involved in responses to hormones and environmental factors as well as cell differentiation and organ development [28,29]. Analysis of cis-acting elements within the promoter region of the ANR genes showed that most cis-acting elements were associated with the MeJA response in *G. barbadense*. Some studies have identified JAV1 as a negative regulator of the jasmonate pathway [30]. When plants are infected by pathogenic bacteria, JA accumulates, initiates JAV1 degradation through the 26S proteasome, activates gene expression, and improves plant disease resistance [31]. JA spraying or increasing the internal JA content can improve the disease resistance of plants [32]. SA acts as a signal molecule in the plant disease resistance response, and when plants are infected by pathogenic microorganisms, the formation of SA is induced [33]. These results suggested that the *GbANR-47* may also be involved in the response to FOV via the MeJA and SA pathway.

Due to limited amounts of arable land, it is difficult to perform crop rotations, which results in annual increases in disease, which has become a challenge in cotton production [34]. At the same time, the main goal of *G. barbadense* breeding is to cultivate new varieties with FOV resistance, high yield, and high quality [33]. Plant hormones such as ethylene and JA are also involved in regulating metabolic processes such as flavonoid synthesis and lignin deposition, thereby enhancing plant resistance to pathogens; MYB transcription factors play an important role in this process [34,35,36]. In this study, *GbANR-47* was identified via expression analysis as a candidate gene and the promoter region of *GbANR-47* contains two MeJA-responsiveness cis-acting elemen and three MYB binding sites. We silenced *GbANR-47* in the disease-resistant cultivar 06-146 to investigate its role in FOV resistance of cotton. We found that, compared with the controls, silenced plants inoculated with FOV had severe disease symptoms and a significantly higher disease index (Figure 7b,d). On the basis of the proanthocyanidin content, it was found that after *GbANR-47* was silenced, the proanthocyanidin content was decreased significantly compared with that in the TRV::00-transformed plants (Figure 7e). A positive correlation between proanthocyanidin content and disease resistance is known for other plants. The anthocyanin content in the peel of *Camellia oleifera* was significantly positively correlated with the resistance of *Camellia oleifera* anthracnose [15,37]. Together, our results suggested that *GbANR-47*, a positive regulator of FOV resistance in *G. barbadense*, may improve resistance to FOV by regulating proanthocyanidin contents.

## 4. Materials and Methods

### 4.1. Plant Materials

Gene expression analysis under FOV stress was performed with the use of 06-146 (disease resistance, Xinjiang Agricultural University College of Agriculture collection) and Xin Hai 14 (disease susceptible, Xinjiang Agricultural University College of Agriculture collection) varieties. The 06-146 and Xin Hai 14 specimens were planted in the soil. The middle and lower parts of the pots were cut with a knife to ensure that the roots of the cotton were damaged in the seedling stage. An FOV spore suspension (1 × 10^7^ spores/L) was applied near the roots of the cotton seedlings, and each pot was inoculated with 50 mL of the spore suspension. Each material was replicated three times, with 15 plants per replicate. Root tissues from two different materials were collected at 0, 1, 3, 6, 12, 24, and 48 h after treatment. When the cotton seedlings were at the three-leaf stage, 50um/L MeJA and SA were used to spray the leaves, and the treated root tissues were collected at 0, 4, 8, 12, 24, and 48 h.

### 4.2. Identification and Bioinformatics Analysis of the ANR Genes in Cotton

This study used the latest published genome sequence as a reference. Genomic and proteomic data of *G. arboreum*, *Gossypium raimondii*, *G. hirsutum*, and *G. barbadense* were obtained from the COTTONGEN (https://www.cottongen.org/data/download, accessed on 13 January 2022) database [38]. The sequences of ANR genes from *Arabidopsis thaliana* and rice were used as seeds. Protein sequences were identified via BLAST searches of cotton sequences. After removing redundant sequences, BLAST software was used to conduct domain validation of all candidate genes in the NCBI Conserved Domains Database (CDD) (https://www.ncbi.nlm.nih. gov/cdd/, accessed on 13 January 2022) [39]. Furthermore, the sequences of the queried cotton ANR gene family members were confirmed. ExPASy software (http://cn.expasy.org/tools, accessed on 13 January 2022) was used to calculate the number of amino acid residues, relative molecular mass, and theoretical isoelectric point of the ANR proteins in cotton. Subcellular localization prediction of the ANR proteins in *G. barbadense* was performed by EuLoc online software (http://euloc.mbc.nctu.edu.tw/, accessed on 13 January 2022) [40,41].

### 4.3. Phylogenetic and Collinearity Analysis of ANR Gene Family Members in Cotton

ClustalW (with the default settings) in MEGA 7 software was used to conduct multiple sequence alignments of the ANR protein sequences from Arabidopsis, rice, *G. arboreum*, *G. raimondii*, *G. hirsutum*, and *G. barbadense* [42]. A phylogenetic tree was subsequently constructed using the neighbor-joining method based on the results of the sequence alignment, where the bootstrap value was set to 1000. The resulting phylogenetic tree was visualized with the online tool Evolview (https://evolgenius.info/, accessed on 14 January 2022).

### 4.4. Chromosomal Location, Gene Structure, and Motif Analysis of the ANR Gene Family 

#### Members in Cotton

The chromosomal location information of ANR gene family members was extracted from island cotton genome annotation files. Chromosome mapping of ANR genes was performed using MapChart software [43]. An evolutionary tree of the ANR genes was constructed by MEGA 7 software, and nwk files were obtained [42]. Motif analysis was performed by the MEME program (in which the number of functional domains was set to 10) [44]. The resulting xml files obtained after running the MEME program, the nwk files of the evolutionary tree, and the gff files of the gene structure were processed and visualized using TBtools software [45].

### 4.5. Analysis of Cis-Acting Elements Upstream of the G. barbadense ANR Gene

The 2000 bp DNA sequence upstream of the ANR gene in *G. barbadense* was extracted using the PlantCARE database (https://bioinformatics.psb.ugent.be/webtools/plantcare/html/, accessed on 14 January 2022), and cis-acting elements were predicted and visualized using the R language ggplot2 package.

### 4.6. RNA Sequencing (RNA-seq) Analysis

Based on the previous research results, standard analysis of the transcriptomes of the disease-susceptible Xin Hai 14 line, the disease-resistant 06-146 line, and a recombinant inbred line (RIL) (Xin Hai 14 crossed with an F_2:7_ 06-146 hybrid) was performed via the raw data to determine expression levels [3]. Then, an expression heatmap was generated using TBtools software [45].

### 4.7. qRT–PCR

According to the cDNA information of the ANR gene in *G. barbadense*, primers were designed for the 5′ and 3′ ends of the gene sequence using Primer 5.0 software (Table S2). The expression of candidate genes was detected via qRT–PCR, in which root stems and leaves tissue cDNA was used as a template, and three replicates were included for each sample. The reference gene was *GbUBQ7*. A Total RNA Extraction Kit (Tiangen, China) was used, and reserve transcription was performed using an M-MLV RTase cDNA Synthesis Kit (TaKaRa, Kyoto, Japan). Real-time PCR amplification was carried out on a Bio-Rad CFX96 Real-time System. An iTaq Universal SYBR Green SuperMix (Bio-Rad, Hercules, CA, USA) kit was used, and the total reaction volume was 20 µL. The reaction procedure was 94 °C predenaturation for 30 s, 95 °C denaturation for 5 s, 60 °C annealing for 5 s, and 72 °C extension for 55 s, of which there were 40 cycles. The results were quantitatively analyzed by using the 2^–ΔΔCt^ method [46].

### 4.8. VIGS

VIGS was performed in cotton based on the tobacco rattle virus (TRV) [47]. First, pTRV1, pTRV2, and pTRV2 containing specific regions of *Gb_ANR-47* were transformed into Agrobacterium strain GV3101. The primers used for the amplification of the *Gb_ANR-47* fragment are shown in Table S1. Ten-day-old seedlings of resistant material 06-146 were transformed with Agrobacterium cultures containing pTRV1 and pTRV2 or a combination of their derived plasmids. After inoculation, 06-146 seedlings were washed with deionized water to remove any excess Agrobacterium inocula and then grown under a 16 h/8 h light/dark photoperiod at 25 °C in an environmentally controlled growth chamber. After injection of a pTRV1 + pTRV2:leaf bleaching of CLA solution in the cotton seedlings, V991 was inoculated, and the incidence of poorly growing plants was monitored for 21 days. At least 15 seedlings were treated in each experiment. A disease severity index (DSI) was calculated for each seedling.

### 4.9. Determination of Anthocyanin Content

After the sample was fixed in an oven at 105 °C for 30 min, it was dried at 80 °C for 2 h until the weight did not change. The dried samples were crushed and passed through a 30–50 mesh sieve, 0.05 g was weighed, 500 μL of extract was added, and the ultrasonic extraction method was used for extraction according to the instructions of the Solarbio Micro Plant Procyanidin Content (OPC) Detection Kit. The standard substance was used to make a standard curve, and the formula y (OPC) = 0.0644 + 0.4529x (measured value) R^2^ = 0.995 was obtained. We used 40 μL of the extract + 160 μL of water as the control group and 40 μL of the extract + 160 μL of the working solution as the measurement group. After a 30-min water bath at 30 °C, the A500 was measured immediately, and the measured value was calculated according to the formula ΔA measurement = A measurement − A control. The measured value was brought into the formula y (OPC) = 0.0644 + 0.4529x, and the content of OPC was calculated.

## 5. Conclusions

In this study, 69 ANR genes were identified through the genome-wide identification of ANR genes in *G. barbadense*, the number of which was significantly greater than the number in *Arabidopsis thaliana* and rice. The phylogenetic results show that the ANR gene family members can be divided into five subgroups and have been relatively conserved during cotton evolution. *GbANR-47* was identified by promoter analysis, expression analysis, and VIGS as a positive regulatory gene in response to FOV stress. This is the first systematic analysis of the ANR gene family and provides a new understanding of FOV resistance in *G. barbadense*. Overall, this study lays a foundation for in-depth functional studies and breeding applications of *GbANR-47*.

## Figures and Tables

**Table 1:** Information on the cotton ANR gene family members.

Gene Name	Gene ID	Open Reading Frame/bp	Protein Length/aa	Relative Molecular Weight(r)/kDa	Theoretical Isoelectric Point (pI)	Subcellular Localization
GbANR-01	Gb_A01G0696	1062	353	38.66	5.65	cytoplasm
GbANR-02	Gb_A01G2252	672	223	24.64	7.54	nucleus
GbANR-03	Gb_A02G1539	900	299	33.45	5.31	chloroplast
GbANR-04	Gb_A02G1557	975	324	35.44	8.71	cytoplasm
GbANR-05	Gb_A02G1593	996	331	36.10	6.36	cytoplasm
GbANR-06	Gb_A05G0439	987	328	35.84	5.83	cytoplasm
GbANR-07	Gb_A05G0715	984	327	35.62	5.77	cytoplasm
GbANR-08	Gb_A05G1219	1017	338	37.23	6.33	cytoplasm
GbANR-09	Gb_A05G1702	1011	336	36.27	5.54	cytoplasm
GbANR-10	Gb_A05G1949	1068	355	39.65	5.67	cytoplasm
GbANR-11	Gb_A05G2812	453	150	16.27	8.84	extracellular space
GbANR-12	Gb_A05G2814	960	319	35.12	6.66	cytoplasm
GbANR-13	Gb_A05G3871	1104	367	41.08	6.06	nucleus
GbANR-14	Gb_A06G0087	1056	351	39.04	5.45	cytoplasm
GbANR-15	Gb_A07G0794	1035	344	38.50	6.48	nucleus
GbANR-16	Gb_A07G1334	726	241	25.89	8.10	organelle membrane
GbANR-17	Gb_A07G1335	990	329	35.89	6.46	cytoplasm
GbANR-18	Gb_A07G1511	1020	339	36.68	7.04	endomembrane system
GbANR-19	Gb_A07G1592	990	329	36.12	5.55	cytoplasm
GbANR-20	Gb_A07G1593	891	296	32.25	5.02	cytoplasm
GbANR-21	Gb_A07G1594	1230	409	46.21	9.22	endomembrane system
GbANR-22	Gb_A07G1596	984	327	35.88	5.94	cytoplasm
GbANR-23	Gb_A08G1080	897	298	33.21	5.02	cytoplasm
GbANR-24	Gb_A08G1297	1116	371	41.31	5.68	nucleus
GbANR-25	Gb_A08G1918	918	305	34.32	5.34	nucleus
GbANR-26	Gb_A08G2417	966	321	35.37	5.62	endomembrane system
GbANR-27	Gb_A09G0335	1083	360	39.91	6.04	cytoplasm
GbANR-28	Gb_A09G1409	936	311	34.18	8.38	cytoplasm
GbANR-29	Gb_A11G0776	1899	632	70.31	5.88	cytoplasm
GbANR-30	Gb_A11G0849	744	247	27.51	8.57	cytoplasm
GbANR-31	Gb_A11G1008	1017	338	36.70	5.81	cytoplasm
GbANR-32	Gb_A12G0107	975	324	36.05	5.89	cytoplasm
GbANR-33	Gb_A12G0108	306	101	10.96	8.78	extracellular space
GbANR-34	Gb_A12G0520	972	323	35.73	6.66	cytoplasm
GbANR-35	Gb_A12G2641	1041	346	37.76	6.51	plasma membrane
GbANR-36	Gb_D01G0734	960	319	34.87	5.85	nucleus
GbANR-37	Gb_D01G2338	972	323	35.86	5.79	endomembrane system
GbANR-38	Gb_D03G0433	996	331	36.05	7.12	cytoplasm
GbANR-39	Gb_D03G0545	906	301	33.94	6.02	nucleus
GbANR-40	Gb_D03G0546	981	326	35.66	5.73	organelle membrane
GbANR-41	Gb_D03G0549	975	324	35.45	8.94	cytoplasm
GbANR-42	Gb_D03G0557	900	299	33.42	5.40	chloroplast
GbANR-43	Gb_D04G0591	1077	358	39.86	7.56	chloroplast
GbANR-44	Gb_D05G0439	990	329	35.96	5.39	cytoplasm
GbANR-45	Gb_D05G0704	984	327	35.60	5.77	cytoplasm
GbANR-46	Gb_D05G1207	1017	338	37.19	6.33	cytoplasm
GbANR-47	Gb_D05G1725	1011	336	36.34	5.55	cytoplasm
GbANR-48	Gb_D05G1976	1068	355	39.75	5.41	cytoplasm
GbANR-49	Gb_D05G2804	897	298	33.41	8.89	mitochondrion
GbANR-50	Gb_D05G2805	960	319	35.06	6.61	cytoplasm
GbANR-51	Gb_D06G0087	1056	351	38.94	5.34	cytoplasm
GbANR-52	Gb_D07G0801	1035	344	38.36	5.98	nucleus
GbANR-53	Gb_D07G1332	573	190	21.55	7.68	cytoplasm
GbANR-54	Gb_D07G1333	990	329	35.79	7.04	cytoplasm
GbANR-55	Gb_D07G1524	1020	339	36.64	6.78	endomembrane system
GbANR-56	Gb_D07G1612	1785	594	64.74	4.92	cytoplasm
GbANR-57	Gb_D07G1616	720	239	26.62	6.08	cytoplasm
GbANR-58	Gb_D07G1617	984	327	35.72	6.46	cytoplasm
GbANR-59	Gb_D08G1045	882	293	33.14	5.02	cytoplasm
GbANR-60	Gb_D08G1399	1035	344	38.30	5.54	endomembrane system
GbANR-61	Gb_D08G1912	918	305	34.41	5.48	cytoplasm
GbANR-62	Gb_D08G2407	972	323	35.58	5.94	cytoplasm
GbANR-63	Gb_D09G0306	1083	360	40.00	5.97	cytoplasm
GbANR-64	Gb_D11G0798	1899	632	70.34	6.56	cytoplasm
GbANR-65	Gb_D11G0879	669	222	24.52	6.45	cytoplasm
GbANR-66	Gb_D11G1037	927	308	33.72	5.58	cytoplasm
GbANR-67	Gb_D12G0116	1008	335	37.19	6.16	cytoplasm
GbANR-68	Gb_D12G0517	846	281	30.91	6.93	cytoplasm
GbANR-69	Gb_D12G2647	1044	347	37.92	6.75	endomembrane system

## Data Availability

Not applicable.

## Supplementary Materials

The following are available online at https://www.mdpi.com/article/10.3390/plants11151902/s1, Figure S1: distribution of the ANR domain in the ANR proteins of cotton; Figure S2: conservative motifs of ANR gene family in *G. barbadense*; Table S1: cis-acting elements analysis of ANR family members in *G. barbadense*; Table S2: all primers used in this study.
