# Supplementary material for: Gb_ANR-47 Enhances the Resistance of Gossypium barbadense to Fusarium oxysporum f. sp. vasinfectum (FOV) by Regulating the Content of Proanthocyanidins
Source: Plants (Basel). 2022 Jul 22;11(15):1902. doi: 10.3390/plants11151902 (PMC9332461; doi:10.3390/plants11151902)
Supplement: Supplementary file 1 [file plants-11-01902-s001.zip › Fig S1.pdf]

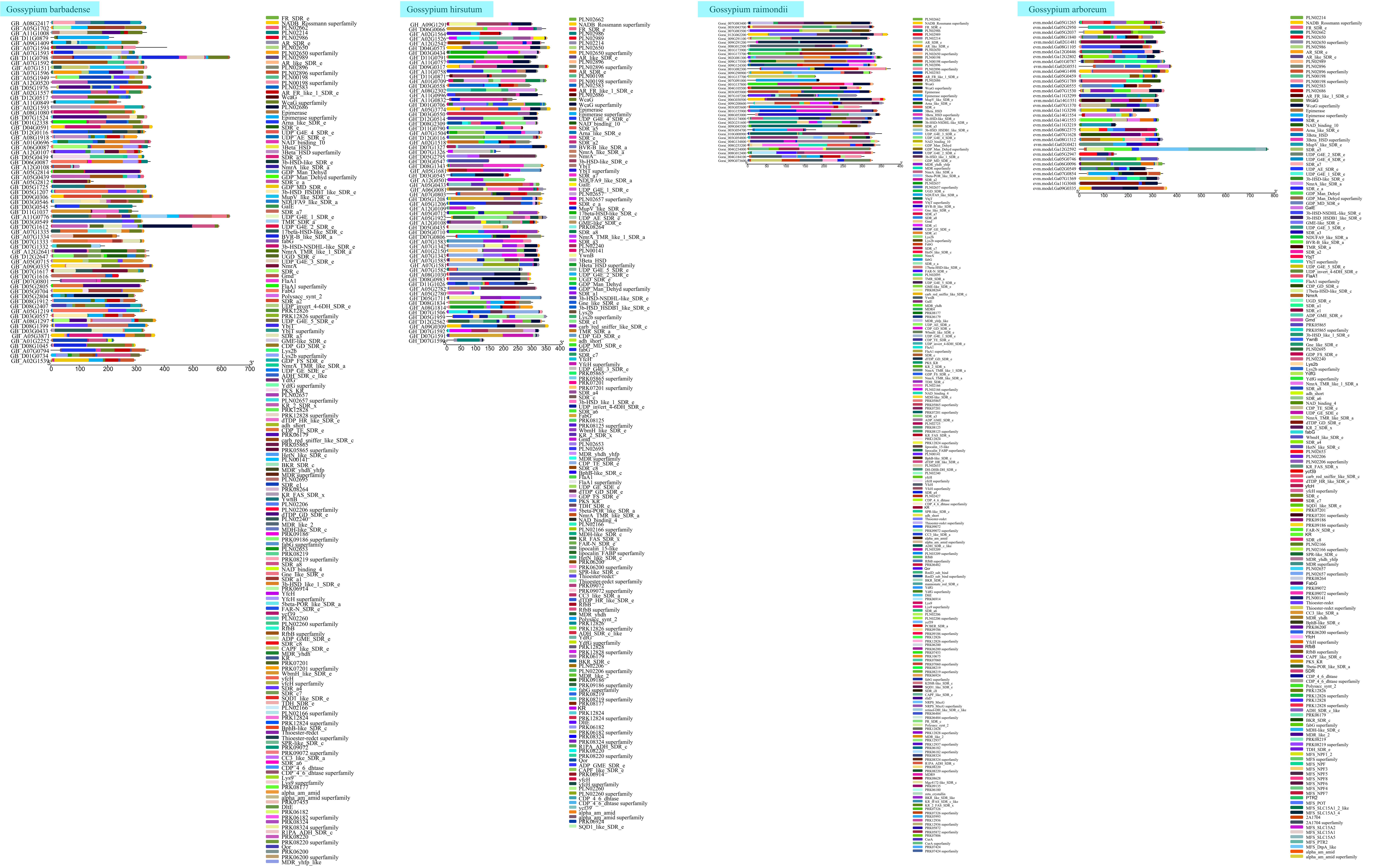

Figure S1.Distribution of the ANR domain in the ANR proteins of cotton.
