# Supplementary figures and images for: Gb_ANR-47 Enhances the Resistance of Gossypium barbadense to Fusarium oxysporum f. sp. vasinfectum (FOV) by Regulating the Content of Proanthocyanidins
Source: Plants (Basel). 2022 Jul 22;11(15):1902. doi: 10.3390/plants11151902 (PMC9332461; doi:10.3390/plants11151902)

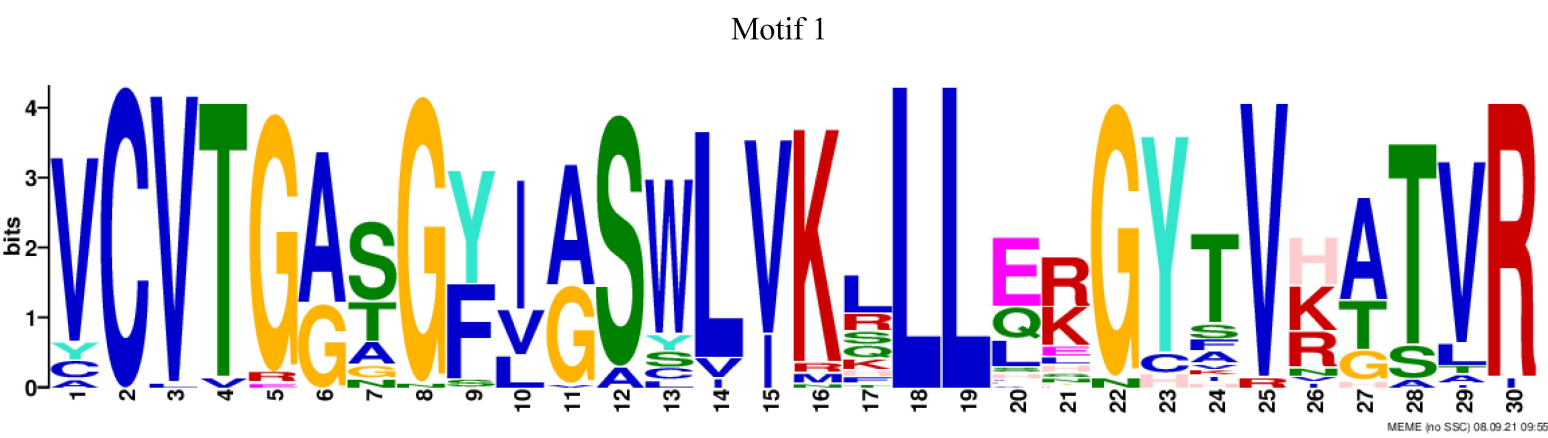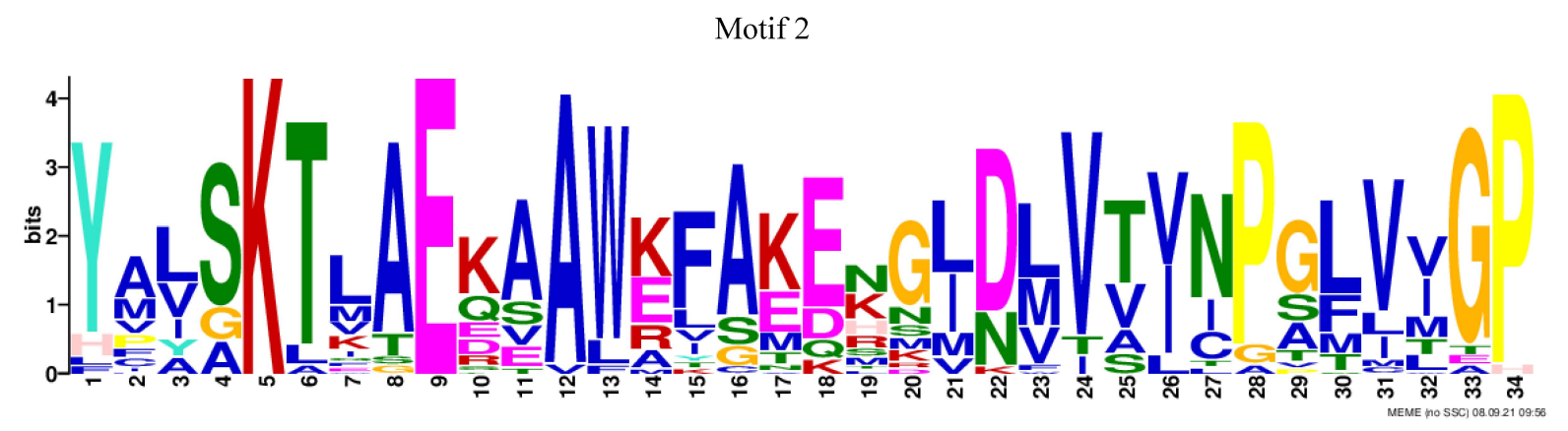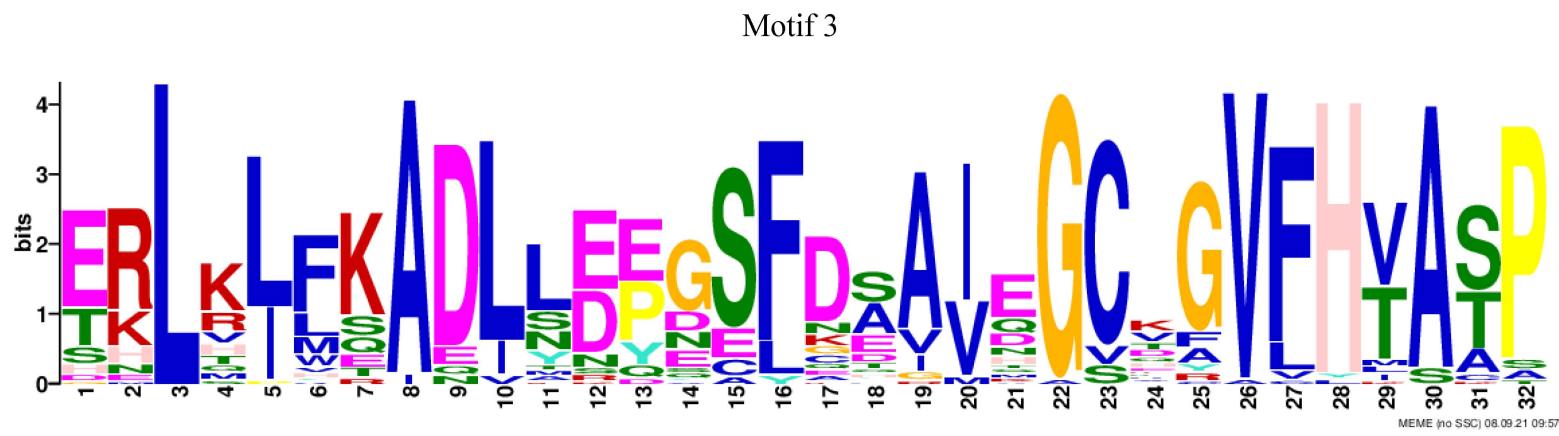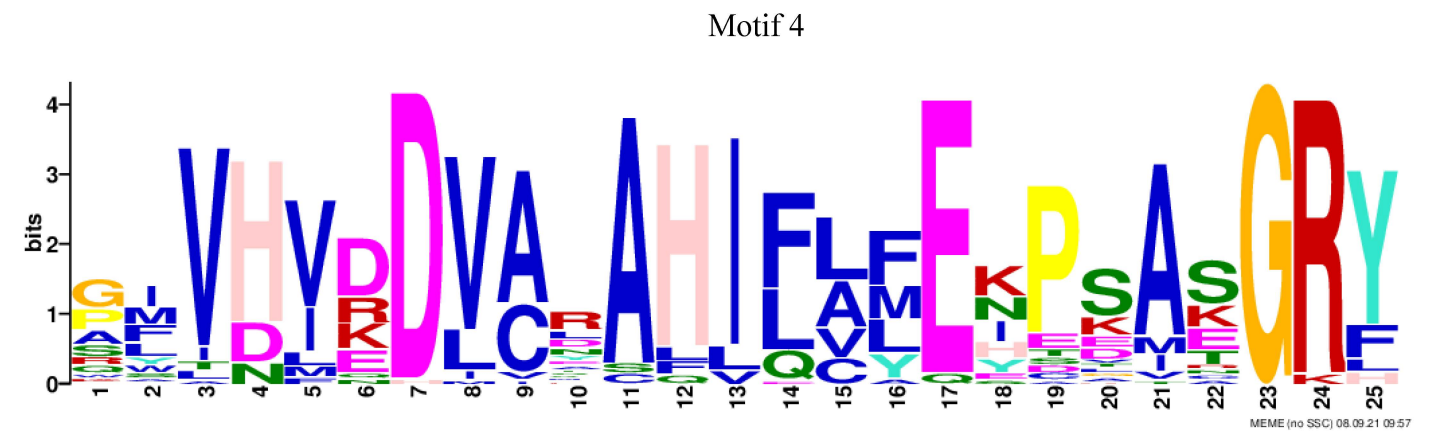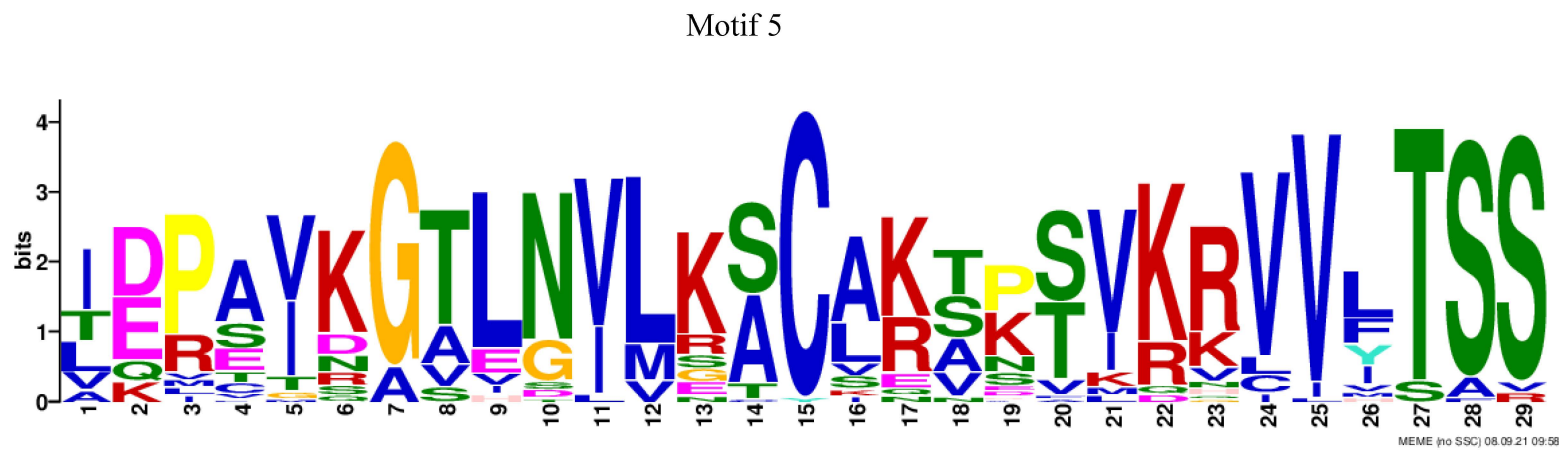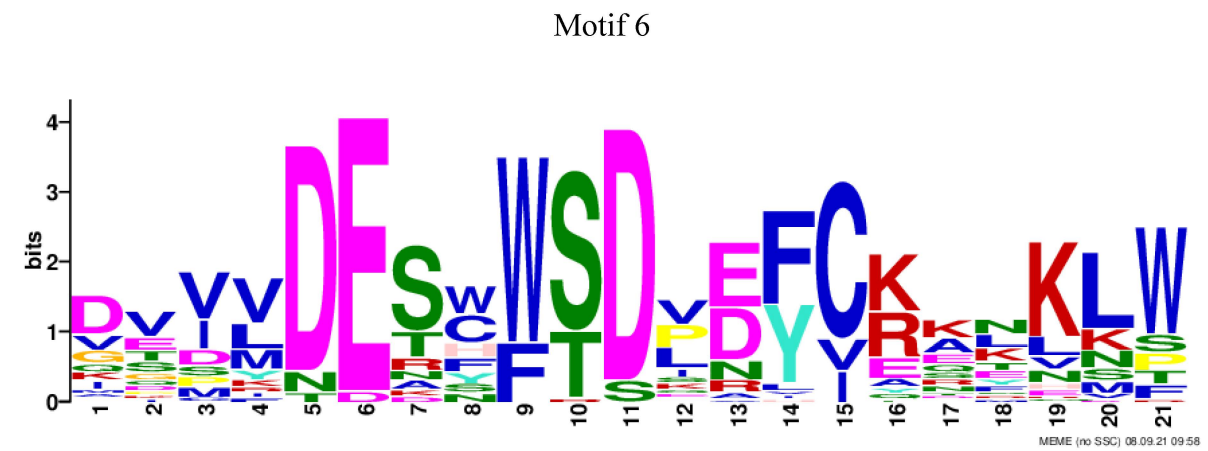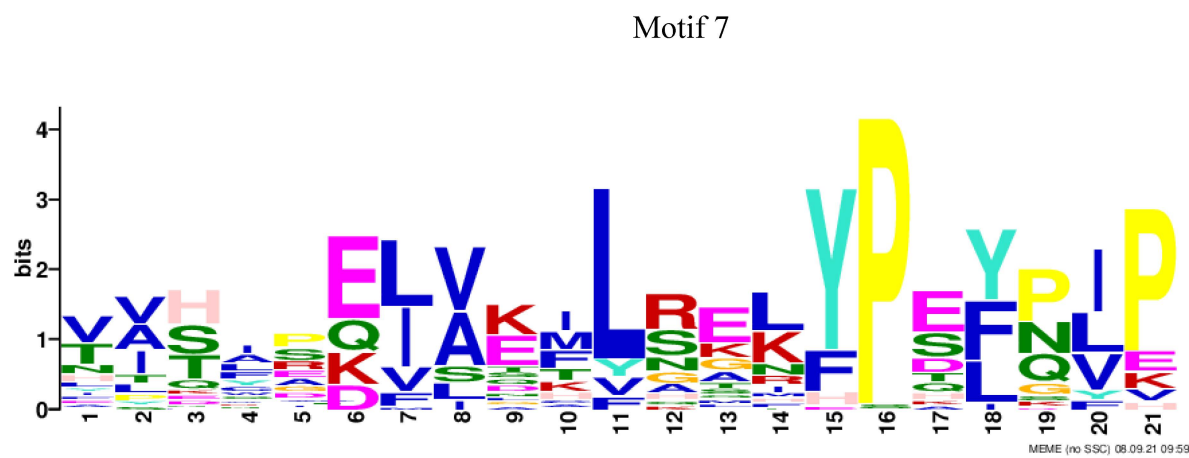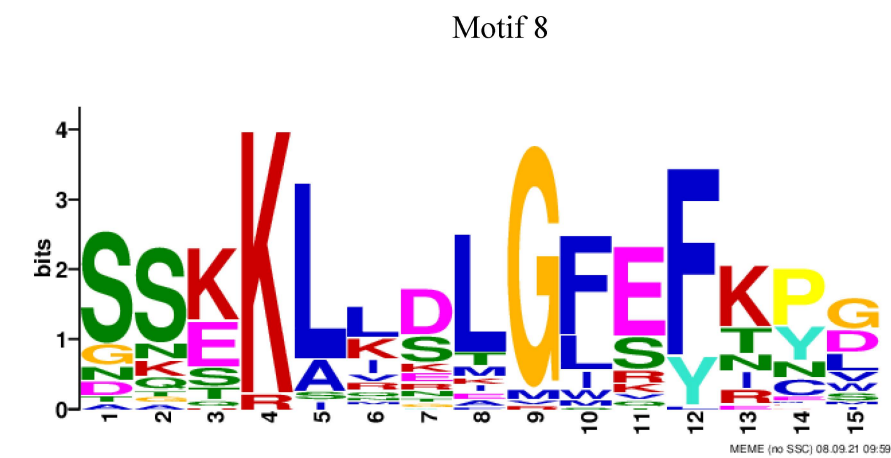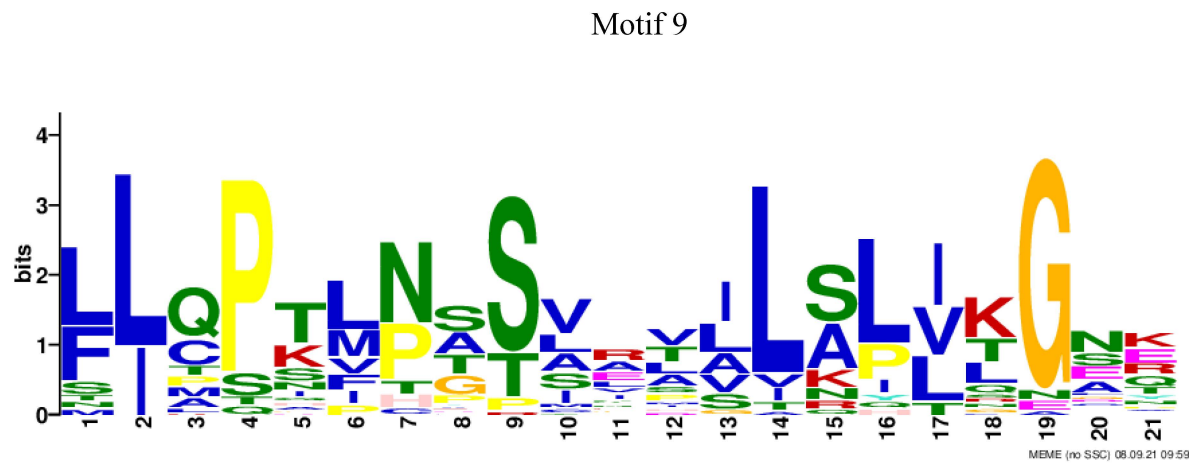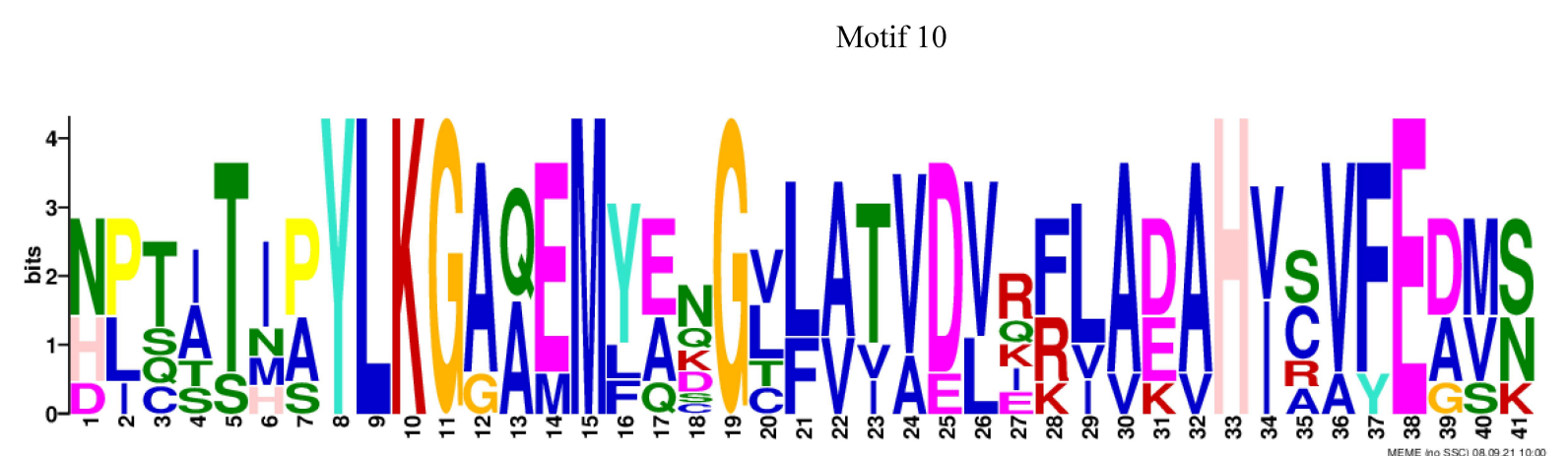

Figure S2. Conservative motifs of ANR gene family in *G. barbadense*

Supplement: Supplementary file 1 [file plants-11-01902-s001.zip › Fig S2.pdf]
